# Association of VDR gene variant rs2228570-*Fok*I with gestational diabetes mellitus susceptibility in Arab women

**DOI:** 10.1016/j.heliyon.2024.e32048

**Published:** 2024-05-28

**Authors:** Maysa Alzaim, Mohammed G.A. Ansari, Abeer A. Al-Masri, Malak N.K. Khattak, Abir Alamro, Amani Alghamdi, Amal Alenad, Majed Alokail, Omar S. Al-Attas, Ahmad G. Al-Zahrani, Nasser M. Al-Daghri

**Affiliations:** a Nutrition Department School of Public Health & Health Sciences. University of Massachusetts, Amherst, MA, 01003, USA; b Chair for Biomarkers of Chronic Diseases, Department of Biochemistry, College of Science, King Saud University, Riyadh, 11451, Saudi Arabia; c Department of Physiology, College of Medicine, King Saud University, Riyadh, 11451, Saudi Arabia; d Department of Biochemistry, College of Science, King Saud University, Riyadh, 11451, Saudi Arabia; e Protein Research Chair, Department of Biochemistry, College of Science, King Saud University, Riyadh, 11451, Saudi Arabia

**Keywords:** Insulin resistance, Hyperglycemia, GDM, VDR gene polymorphism, Saudi pregnant women, SNP

## Abstract

Gestational diabetes mellitus (GDM) has been linked with adverse pregnancy outcomes. Vitamin D receptor (VDR) gene variants have been associated with diabetes mellitus susceptibility and related complications. This study assessed the association between VDR gene polymorphism (rs2228570) and GDM risk among pregnant Arab women. A total of 368 pregnant Saudi women who were screened for GDM at 24−28 weeks of gestation and genotyped for the VDR gene variant (rs2228570) were included in this cross-sectional study. Circulatory insulin levels, fasting blood glucose (FBG), glycated hemoglobin (HbA1c), and vitamin D (25(OH)D) were measured. There were 108 women with GDM and 260 women without GDM. The genotype frequency of women with GDM was CC 60.2 %, CT 33.3 %, TT 6.9 %, and CT + TT 39.8 %; for non-GDM women, were CC 61.1 %, CT 31.5 %, TT 6.9 %, and CT + TT 38.4 %. No association was found between the VDR gene variant (rs2228570-*Fok*I) and GDM susceptibility after adjustment for covariates. Serum 25(OH)D had a significant inverse association with FBG (r = −0.49, p = 0.01) and HbA1c (r = −0.45, p = 0.03) among carriers of the TT-genotype. Furthermore, a significant inverse correlation was observed between serum 25(OH)D and HOMA-β (r = −0.20, p = 0.035) in individuals with the T-allele. Among pregnant Saudi women, glycemic indices appear to be influenced by vitamin D, suggesting a possible role it may play in mitigating the metabolic changes associated with GDM, particularly among individuals with specific genetic backgrounds. In our study population, rs2228570-*Fok*I did not appear to be a significant contributor to GDM risk.

## 1 Introduction

Gestational diabetes mellitus (GDM) is a common endocrine disorder that usually manifests during the second or third trimester of pregnancy [1]. It is characterized by glucose intolerance or hyperglycemia of varying severity, detrimental effects of which can be acute or long term on both the health of the mother and infant [[2], [3], [4]]. The global prevalence of GDM ranges from 7.1 % to 27.6 %, depending on the geographical location, ethnicity, and the approaches used to screen and diagnose GDM [[5], [6], [7]]. Individuals with GDM have a greater risk of developing maternal cardiovascular disease, hypertension, macrosomia, pre-eclampsia, the need for Cesarean delivery, and premature birth [[8], [9], [10]]. In addition, they have a seven-fold increased risk of developing type 2 diabetes mellitus (T2DM) if left untreated [11,12], and are 35–80 % more likely to suffer from GDM in succeeding pregnancies [13]. Furthermore, the children of GDM pregnancies are more likely to become obese and/or develop T2DM in their early years [[13], [14], [15]].

Globally, around 46%–87 % of pregnant women are vitamin D [25(OH)D] deficient (serum 25(OH)D < 50 nmoL/l) [16]. The prevalence rate of vitamin D deficiency in Saudi pregnant women as of 2016 has reached 87 % [[17], [18], [19]]. Though the GDM etiology has not been fully elucidated, several studies consider GDM as a multifactorial condition where genetics, lifestyle, and environment are causative factors for its progression [[20], [21], [22]]. Furthermore, maternal vitamin D deficiency has been identified as one of the risk factors for the onset of GDM [23,24]. Studies have also shown that the vitamin D metabolites and its receptors play a significant role in insulin secretion and sensitivity [25]. The vitamin D receptor belongs (VDR) to the nuclear receptor superfamily of transcriptional regulators and plays a significant role in 1,25(OH)2D signaling [26].

VDR gene variants have recently gained attention and are associated with susceptibility to various clinical conditions [[27], [28], [29]]. Most VDR gene variants (*Bsm*I*, Apa*I*, and Taq*I) are located at the 3′ untranslated regions (3′ UTR). In contrast, the rs2228570 (*Fok*I) is a common polymorphism located within the 5′ end of the gene, near the promoter region [30]. Numerous studies have associated VDR gene polymorphisms with the risk of several health impediments, including bone disorders, cancer and autoimmune diseases [28,29,31]. Moreover, variations in *Fok*I has functional implications on vitamin D signaling pathways.

VDR gene polymorphisms have also been implicated in GDM pathogenesis, although results were inconsistent [[32], [33], [34]]. Investigating this variant may reveal insights how vitamin D impacts health outcomes, GDM in particular. In the VDR gene variant (rs2228570-*Fok*I), the T-allele causes a nucleotide change for the gene sequence, forming a second upstream start site and culminating in VDR allele expression [35,36]. This longer VDR has lower response levels to 1,25(OH)2D than the shorter VDR in activating target gene expressions [37]. We hypothesize that Saudi women with low 25(OH)D levels and this longer, less efficient T-allele of the VDR gene are at a higher risk for 25(OH)D deficiency and GDM. There is a paucity of studies exploring the relationship between VDR gene variants and GDM susceptibility, particularly in the Arab population. Hence, the current study aimed to explore the link between the VDR gene variant (rs2228570-*Fok*I) and GDM susceptibility.

## 2 Materials and methods

### 2.1 Study design and participants

This study cohort included 368 pregnant Saudi women who visited antenatal clinics during the second trimester of their pregnancy (24−28 weeks) at King Khalid University Hospital (KKUH), King Fahad Medical City (KFMC) and King Salman bin Abdulaziz Hospital, all in Riyadh, Saudi Arabia. All participants submitted a signed informed consent.

This study received approval from the Ethics Committee of the College of Medicine at King Saud University (KSU), Riyadh, Saudi Arabia, under approval number E−13-1013, dated February 11, 2014. All methods and protocols were conducted in accordance with relevant guidelines and regulations, adhering to the principles of the Declaration of Helsinki.

### 2.2 Inclusion criteria

Eligible participants were pregnant Saudi women aged 18−40 with no history of diabetes (type 1 or 2) before the 16th week of their pregnancies.

### 2.3 2.3Exclusion criteria

Participants with a gestational age <16 weeks, were on vitamin D supplements, were taking oral glucocorticoids or other drugs known to disrupt the vitamin D or calcium absorption, individuals with parathyroid disorders; had chronic medical ailments, or preexisting liver or kidney conditions, or chronic severe diseases such as epilepsy, cancer, or other malignancy, were excluded.

### 2.4 Interview questionnaire

Clinical data were taken during their GDM screening visit (24−28 weeks). Participants were asked about their risk factors for GDM, including a history of miscarriage, family history of diabetes, and parity.

### 2.5 Anthropometric measurements

Baseline height (cm) and weight (kg) were measured (standing, upright, barefoot) to the closest 0.5 cm and 0.1 kg, respectively, using a digital Pearson scale (ADAM Equipment Inc., USA). Pre-pregnancy body mass index (BMI) was calculated (kg/m^2^) based on self-reported heights and pre-pregnancy weights.

### 2.6 Blood collection and biochemical assessment

Fasting blood samples (10 mL) were extracted during their GDM screening visit using a sterile vacutainer blood collection system. Each sample was aliquoted and stored in a freezer at −80 °C for subsequent analysis. The Chair for Biomarkers of Chronic Diseases (CBCD) at KSU, was responsible for storing and analyzing all samples. Glycated hemoglobin (HbA1c) levels were measured from whole-blood samples using a point-of-care instrument (Accu-Check Active, Roche Diagnostics GmbA, Mannheim, Germany), while fasting insulin levels were measured using the COBAS e411 Analyzer (Roche Diagnostics GmbA, Mannheim, Germany). Insulin resistance and basal pancreatic β-cell function, homeostasis model assessment of β-cell function (HOMA-β), and HOMA-IR were calculated using the equations below:

HOMA-IR = fasting insulin (μU/ml) × fasting blood glucose (FBG) (mmol/L)/22.5 [38].

HOMA-β = 20 × fasting insulin (μU/ml)/ [FBG (mmol/L) − 3.5] [38].

Serum 25(OH)D levels were assessed using Electrochemiluminescence Binding Assay kits (ECLIA) from Roche Diagnostics GmbA, Mannheim, Germany. The coefficients of variation (CV) for inter- and intra-assay were 5.3 and 4.6, respectively. For 25(OH)D, enzyme-linked immunosorbent assay (ELISA), with 75 % cross-reactivity to 25(OH)D2 and 100 % cross-reactivity to 25(OH)D3 was used as done in previous studies [39,40]. Vitamin D deficiency was defined as serum 25(OH)D below 50 nmol/L [41].

### 2.7 GDM screening

All participants were screened for GDM using the International Association of Diabetes and Pregnancy Study Groups (IADPSG) guidelines which use the following criteria: FBG ≥5.1 mmol/L and/or 1 h post-glucose load ≥10 mmol/L and/or 2 h post-glucose load ≥8.5 mmol/L [37].

### 2.8 Genotyping

From the whole-blood samples, genomic DNA was isolated using and following the innuPREP blood mini kits (Analytik Jena, Germany) and guidelines provided by the manufacturer, respectively. The concentration of purified DNA was quantified using the Nanodrop spectrophotometer (ND-1000, Nanodrop Technologies, Wilmington, DE, USA). *Fok*I SNP (rs2228570) was assessed via real-time polymerase chain reaction (RT-PCR) allelic-discrimination analysis employing the pre-designed TaqMan genotype assay supplied by Applied Biosystems, Foster City, CA, USA (assay ID: C_12060045_20). Our previous work mentioned the detailed protocol [27,28]. The Biorad CFX manager software and methodology outlined in our earlier research followed in achieving the Genotype assignments [42].

### 2.9 Statistical analysis

Data to perform power analysis was taken from a previous study by Aslani et al. [43]. G*Power Calculator was used for GDM and healthy subjects in which TT genotype was more common in GDM (odd ratio = 1.783), probability of TT = 0.062, Alpha error of probability = 0.05 and Power (1-β) = 0.85 (reference: VDR *Fok*I polymorphism and its potential role in the pathogenesis of gestational diabetes mellitus and its complications) the total sample size was achieved = 343 with actual power = 0.8501. Data were analyzed using SPSS version 21.0, IBM. Normality tests were conducted for all quantitative variables using the Shapiro−Wilk Test. Quantitative variables with normal distribution were presented as mean ± standard deviation (SD), while those with non-normal distributions were presented as median (25th and 75th percentiles). Categorical data were presented by frequencies and percentages (%) and differences in categorical variables were determined using the Chi-square test. Independent T-test (for normal variables) and Mann-Whitney *U* Test (for non-normal variables) were used to compare continuous variables. Correlation analyses were done to determine relationships between variables of interest. Logistic regression analysis was used to determine unadjusted and adjusted risk. Generalized multivariate analysis was also performed to compare mean differences adjusted for covariates BMI and age. Finally, a p-value <0.05 was considered statistically significant.

## 3 Results

### 3.1 Clinical characteristics of subjects

**Fig. 1:**
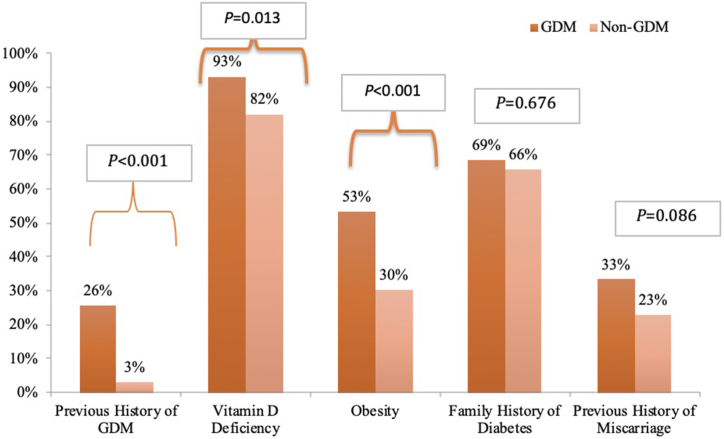
Demographic and biochemical parameters in relation to GDM status.

Table 1 shows the biochemical and anthropometric characteristics of subjects with and without GDM. Subjects with GDM had significantly higher BMI and were older than controls (p-values <0.001 and < 0.001, respectively). Furthermore, 53.4 % of the participants with GDM were obese, compared to only 30.2 % in the control group (p < 0.001) (Fig. 1). As expected, the GDM group had significantly higher fasting insulin levels, FBG, post-glucose loads, HbA1c, HOMA-IR and HOMA-β (p-values <0.001) than controls. No differences were seen in mean 25(OH)D levels. However, the GDM group had a significantly higher prevalence of vitamin D deficiency (93 %) than controls (p = 0.01). Additionally, the GDM group also had a higher prevalence of previous GDM than controls (25.7 % vs 3.1 %, p < 0.01) (Fig. 1).

**Table 1:** Demographic and biochemical characteristics of the subjects with GDM and the healthy subjects.

Parameters	All	GDM	Control	P-value	P-value*
N	368	108 (29.3)	260 (70.7)		
Age (years)	29.1 ± 5.6	30.6 ± 6.0	28.4 ± 5.2	<0.001	
Parity	2.0 (1.0–4.0)	2.0 (1.0–5.0)	2.0 (1.0–3.0)	0.37	0.17
BMI (kg/m^2^)	28.2 ± 6.1	30.3 ± 6.4	27.3 ± 5.9	<0.001	–
Pre-pregnancy BMI (kg/m^2^)	26.9 ± 5.9	28.7 ± 5.9	25.9 ± 5.4	<0.001	–
FBG (mmol/L)	4.5 (4.1–5.0)	5.3 (4.8–5.6)	4.3 (3.9–5.6)	<0.001	<0.001
OGTT 1 h (mmol/L)	7.4 (5.8–8.9)	10.1 (7.6–10.7)	6.7 (5.3–7.8)	<0.001	<0.001
OGTT2h (mmol/L)	6.3 (5.3–7.7)	8.8 (6.6–10.2)	5.9 (5.1–6.9)	<0.001	<0.001
HbA1c (%)	4.8 ± 0.5	5.0 ± 0.6	4.7 ± 0.5	<0.001	<0.001
Insulin (uU/ml)	7.5 (4.5–13.1)	9.7 (6.2–17.2)	6.5 (4.1–12.1)	<0.001	0.003
HOMA-IR	1.5 (0.9–2.5)	2.2 (1.3–3.9)	1.2 (0.7–2.2)	<0.001	<0.001
HOMA-β	141.9 (53–326)	371 (163–659)	100 (45–216)	<0.001	<0.001
25(OH)D (nmol/l)	33.4 (213–54)	33.9 (22–57)	32.9 (21–53)	0.77	0.69

**Note:** Normally distributed variables are presented as mean ± SD. Non-normally distributed variables such as parity, FBG, OGTT_1 h and 2 h, fasting insulin, HOMA-IR, HOMA-β, and vitamin D are presented as medians (25th and 75th percentiles). *denotes p-value adjusted for age and BMI. Significant at <0.05.

### 3.2 Risk factors for GDM

Table 2 shows the independent risk for GDM and these include identified were the previous history of GDM (OR 9.25, 95 % CI 3.14−27.22, p < 0.001), fasting insulin (OR 1.07, 95 % CI 1.03−1.11, p < 0.001), HOMA-IR (OR 75.34, 95 % CI 15.32−370.60, p < 0.001), obesity (OR 2.08, 95 % CI 1.09−3.95, p = 0.026), and vitamin D deficiency (OR 2.96, 95 % CI 1.22−7.19, p = 0.05).

**Table 2:** Predictors of GDM among the subjects at 24– to 28-week gestation.

Parameters	Univariate analysis		Adjusted model	
	OR (95 % CI)	P-value	OR (95 % CI)^a^	P-value^a^
Age (years)	1.05 (1.00–1.10)	0.04	0.98 (0.91–1.04)	0.45
Parity	1.11 (0.96–1.30)	0.17	0.86 (0.68–1.08)	0.19
Current BMI (kg/m^2^)	1.05 (1.02–1.10)	0.002	1.04 (0.99–1.09)	0.17
Pre-pregnancy BMI (kg/m^2^)	1.06 (1.03–1.10)	0.001	1.05 (0.99–1.11)	0.07
Family history of diabetes	1.13 (0.63–2.00)	0.68	0.84 (0.42–1.65)	0.61
Previous history of GDM	10.96 (4.1–29.0)	<0.001	9.25 (3.1–27.2)	<0.001
History of miscarriage	1.69 (0.93–3.10)	0.09	1.36 (0.64–2.92)	0.43
HbA1c (%)	2.44 (1.51–3.92)	<0.001	1.50 (0.74–3.02)	0.26
Fasting insulin (uU/ml)	1.07 (1.03–1.10)	<0.001	1.07 (1.03–1.11)	<0.001
HOMA-IR	56.8 (12.7–254.6)	<0.001	75.34 (15–370)	<0.001
HOMA-β	0.48 (0.24–0.96)	0.04	0.76 (0.36–1.57)	0.45
25(OH)D (nmol/L)	0.58 (0.19–1.81)	0.35	1.00 (0.98–1.02)	0.97
25(OH)D deficiency (<50 nmol/L)	2.96 (1.22–7.19)	0.05	2.29 (0.79–6.65)	0.22
Obesity	2.66 (1.62–4.36)	<0.001	2.08 (1.09–3.95)	0.03

P-value.

a adjusted for age, pre-pregnancy BMI, family history of diabetes, previous history of GDM, HbA1c (%), and obesity.

### 3.3 *Fok*I VDR polymorphism and GDM

Table 3 showed no significant differences in the *Fok*I genotypes between groups. Using the CC genotype as a reference point, there was no notable variation in GDM risk was observed across different *Fok*I VDR genotypes, even after adjusting for vitamin D deficiency, HbA1c, fasting serum insulin, previous history of GDM, obesity, BMI, age, and family history of diabetes.

**Table 3:** Comparison of the genotype and allele frequencies of the VDR polymorphism rs2228570 (*Fok*I) in the subjects with GDM and the Control subjects.

Parameters	All	GDM	Control	Odds ratio (95 % CI)	P-value	Adjusted OR (95 % CI)	Adjusted p-value
N		N = 108 (29.3 %)	N = 260 (70.7 %)				
**rs10735810 (*Fok*I)**							
**CC**	225 (61.1)	65 (60.2)	160 (61.1)	1		1	
**CT**	118 (32.1)	36 (33.3)	82 (31.5)	0.96 (0.38–2.40)	0.93	1.26 (0.48–3.31)	0.43
***TT***	25 (6.8)	7 (6.5)	18 (6.9)	1.08 (0.66–1.76)	0.76	1.28 (0.76–2.14)	0.29
**CT + TT**	143 (38.9)	43 (39.8)	100 (38.4)	1.06 (0.66–1.68)	0.81	1.29 (0.78–2.07)	0.26
**C**	568 (77.2)	166 (76.9)	402 (77.3)	1		1	
***T***	168 (22.8)	50 (23.1)	118 (22.7)	1.03 (0.70–1.50)	0.70	1.51 (0.78–1.70)	0.37

**Note:** OR: odds ratio (95 % CI), p-value adjusted for age, pre-pregnancy BMI, family history of diabetes, obesity, previous history of GDM, fasting serum insulin, HbA1c, and vitamin D deficiency.

### 3.4 Correlations between vitamin D and other parameters in genotypes

**Fig. 2:**
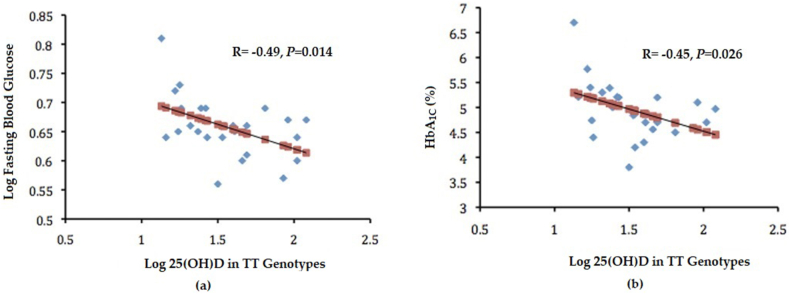
Correlations between log vitamin D (nmol_L) in rs2228570-TT genotypes vs. (a) FBG and (b) HbA 1_C_.

**Fig. 3:**
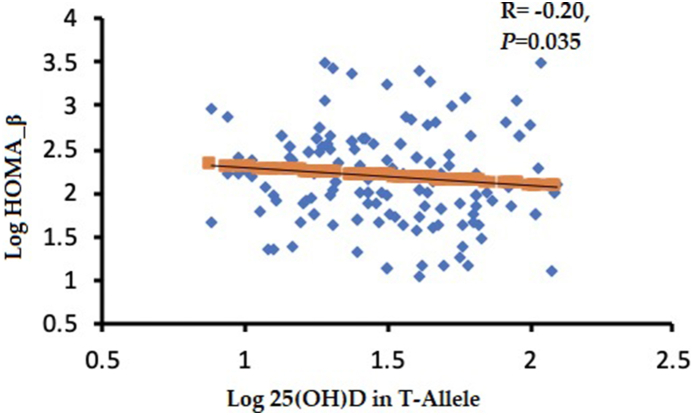
Correlation between log vitamin D (nmol_L) in *T*-allele vs. HOMA-β.

Serum 25(OH)D had a significant inverse association with FBG (r = −0.49, p = 0.01) (Fig. 2A) and HbA1c (r = −0.45, p = 0.03) (Fig. 2B) for carriers of the rs2228570-TT genotype. Furthermore, participants with the T-allele showed a significant inverse association between serum 25(OH)D and HOMA-β (r = −0.20, p = 0.035) (Fig. 3).

## 4 Discussion

The current study assessed the relationship between the VDR gene variant rs2228570 and GDM risk among pregnant Saudi women and showed no significant link between this gene variant (rs2228570) and GDM susceptibility among the Saudi population.

Globally, the prevalence of 25 [OH]D deficiency amongst pregnant women ranges from 1 to 90 %, with the Middle East having the largest inadequacies [44]. Emerging evidence suggests that 25(OH)D supplementation in pregnant women might regulate metabolic alteration, including hyperglycemia by improving insulin sensitivity [43]. 25(OH)D exhibits cellular activities after binding to VDR, a nuclear hormone receptor family member localized on chromosome 12q3.1 and is primarily expressed in the pancreas [28]. Notably, the *Fok*I variant has also been linked to the genetic heterogeneity of T1DM and T2DM [30,45,46]. In T1DM patients, the TT genotype was twice more prevalent than their healthy counterparts [45]. It may potentially aid in T1DM development by weakening insulin production or altering the immunosuppressive effects of vitamin D since the T-allele correlates to a less active VDR protein [47,48].

Several studies explored the susceptibility link between VDR gene variants and GDM. Variations in the VDR gene might impact the GDM risk [32,34,49,50]. VDR gene variants, notably rs2228570, have been associated with altered insulin secretion, glucose metabolism and lower vitamin D levels [33,51]. It has been demonstrated that vitamin D boosts peripheral tissue glucose absorption and modifies pancreatic β-cell release of insulin to increase sensitivity. Consequently, VDR gene variations might cause reduced insulin sensitivity due to altered vitamin D signaling. In fact, several studies have comprehensively cataloged the association of specific VDR gene variants with health impediments like obesity, T2DM, PCOS, and CVD, which also serve as predisposing factors for GDM [29,[52], [53], [54]].

The current study revealed that the rs2228570-*Fok*I variant is not associated with GDM among pregnant Saudi women. Our findings were consistent with other studies on a smaller scale in Saudi Arabia, and Brazil [50,55], in addition, a case-control study in the Chinese population found neither a significant association with GDM risk nor gene-gene interactions was observed among the investigated VDR gene SNPS [56]. On the contrary, other studies have reported the significant contributory role of this VDR polymorphism in GDM pathogenesis [34,43]. However, such studies are limited, and their findings are inconclusive.

Aslani et al. on a cohort of 303 pregnant Iranian women, reported that women with GDM were more likely to have the TT genotype than non-GDM subjects [43]. The C-allele was modestly more common in non-GDM subjects (78.6 % vs 72.2 %; p = 0.06), suggesting that the C-allele may be associated with lower rates of GDM [43]. The researchers proposed that the mutant T-allele has a long structure and is 40 % less active than the C-allele, which puts the carriers of the T-allele at higher risk of developing GDM [43]. A similar study in Turkey [34] supported their finding. Furthermore, a meta-analysis by Liu S [57], confirmed a significant association between the rs2228570 VDR gene variant and GDM in the recessive model in the overall population, further subgroup analysis by race confirmed its significant association in the Caucasian population and suggested that *Fok*I*-* rs2228570 along with ApaI (rs7975232) VDR gene variant could potentially be used as a molecular biomarker in screening and diagnosing GDM [57].

Our study showed no association between GDM risk and *Fok*I VDR polymorphism. However, it showed that carriers of the TT genotype had a significant inverse association between serum 25(OH)D and glycemic indices. It is also known that VDR can modify glucose homeostasis through the insulin-like growth factor system [19,58]. Our study among pregnant Saudi women suggest that vitamin D status might have influenced circulatory glucose levels, which is partially dependent on *Fok*I genotypes. However, the roles that vitamin D and VDR play in regulating glucose homeostasis are not yet very clear.

Our study found no significant differences in vitamin D levels of participants with GDM and those without. Nevertheless, some observations from different populations have indicated a significant correlation between vitamin D levels and GDM [12,49,59,60], suggesting ethnic difference. Nevertheless, our study showed that those who were vitamin D deficient were twice likely to develop GDM than those with normal vitamin D levels, confirming previous observations [[61], [62], [63], [64]]. Several studies on GDM highlighted the significant role of vitamin D in both the functioning of β-cells and impaired glucose tolerance development [65,66]. However, the association is inconsistent since other studies undertaken in Saudi Arabia [67], Turkey [68], the Czech Republic [69], India [70], and Britain [71] did not find any correlation between vitamin D deficiency and the risk of developing GDM. The discrepancies in the outcomes of the previous studies might be attributable to various factors that could have impacted the results, including ethnicity, location, sample size, study design, heterogeneous diets, failure to adjust for confounding factors, and socioeconomic status [65,72,73].

The present study has certain limitations. The cross-sectional design cannot prove causation. Additionally, the small sample size, particularly concerning carriers of the TT genotype of the *Fok*I VDR polymorphism, made it difficult to assess the correlation between GDM risk and VDR gene polymorphisms. Nevertheless, this is the first study in Saudi Arabia to evaluate the correlation between GDM and the *Fok*I VDR polymorphism, considering factors such as the family history of diabetes, previous history of GDM, obesity, parity, vitamin D deficiency, BMI, and age. The present study had more stringent criteria with a larger sample size than previous local studies. Additionally, this study possesses several unique glycemic indices including insulin and HbA1c, which are not available elsewhere. These indices adhered to the globally recognized and cost-effective criteria established by IADSPG [74,75].

## 5 Conclusions

The VDR genetic variant rs2228570 is not associated with GDM risk in this ethnic population. However, glycemic indices appear to be influenced by vitamin D status, particularly among carriers of the TT genotype of *Fok*I VDR. Further research using a diverse statistical approach, enrolling subjects with varying ethnicities and considering both the traditional and non-traditional GDM risk factors along with other genes affecting the vitamin D metabolic pathway is needed. Considering the effect of vitamin D levels on pregnancy outcomes and various diseases beyond GDM, it is recommended that pregnant Saudi women should consider vitamin D supplementation.

## Funding

The authors extend their appreciation to the Deputyship for Research and Innovation, 10.13039/100009950Ministry of Education in Saudi Arabia, funding this research work through project number IFKSUOR3-017-5.

## Institutional review board statement

The study was conducted in accordance with the Declaration of Helsinki and approved by the Ethics Committee of the College of Medicine [Approval no E−13-1013, February 11, 2014], King Saud University (KSU), Riyadh, KSA.

## Informed consent statement

Informed consent was obtained from all subjects involved in the study.

## Data availability statement

Data is available upon request to the corresponding author.

## CRediT authorship contribution statement

**Maysa Alzaim:** Writing – original draft, Conceptualization. **Mohammed G.A. Ansari:** Writing – original draft, Methodology. **Abeer A. Al-Masri:** Writing – review & editing, Methodology, Investigation. **Malak N.K. Khattak:** Writing – review & editing, Formal analysis. **Abir Alamro:** Writing – review & editing, Methodology, Investigation. **Amani Alghamdi:** Writing – review & editing, Project administration, Methodology, Investigation. **Amal Alenad:** Writing – review & editing, Methodology, Investigation. **Majed Alokail:** Writing – review & editing, Supervision, Methodology. **Omar S. Al-Attas:** Writing – review & editing, Supervision. **Ahmad G. Al-Zahrani:** Writing – review & editing, Methodology, Investigation. **Nasser M. Al-Daghri:** Writing – review & editing, Supervision, Resources, Funding acquisition.

## Declaration of competing interest

The authors declare that they have no known competing financial interests or personal relationships that could have appeared to influence the work reported in this paper.
